# Allergen immunotherapy in the current COVID-19 pandemic: A position paper of AeDA, ARIA, EAACI, DGAKI and GPA: Position paper of the German ARIA Group^A^ in cooperation with the Austrian ARIA Group^B^, the Swiss ARIA Group^C^, German Society for Applied Allergology (AEDA)^D^, German Society for Allergology and Clinical Immunology (DGAKI)^E^, Society for Pediatric Allergology (GPA)^F^ in cooperation with AG Clinical Immunology, Allergology and Environmental Medicine of the DGHNO-KHC^G^ and the European Academy of Allergy and Clinical Immunology (EAACI)^H^

**DOI:** 10.5414/ALX02147E

**Published:** 2020-05-28

**Authors:** Ludger Klimek, Oliver Pfaar, Margitta Worm, Karl-Christian Bergmann, Thomas Bieber, Roland Buhl, Jeroen Buters, Ulf Darsow, Thomas Keil, Jörg Kleine-Tebbe, Susanne Lau, Marcus Maurer, Hans Merk, Ralph Mösges, Joachim Saloga, Petra Staubach, Petra Stute, Klaus Rabe, Uta Rabe, Claus Vogelmeier, Tilo Biedermann, Kirsten Jung, Wolfgang Schlenter, Johannes Ring, Adam Chaker, Wolfgang Wehrmann, Sven Becker, Norbert Mülleneisen, Katja Nemat, Wofgang Czech, Holger Wrede, Randolf Brehler, Thomas Fuchs, Peter-Valentin Tomazic, Werner Aberer, Antje Fink-Wagner, Friedrich Horak, Stefan Wöhrl, Verena Niederberger-Leppin, Isabella Pali-Schöll, Wolfgang Pohl, Regina Roller-Wirnsberger, Otto Spranger, Rudolf Valenta, Mübecell Akdis, Cezmi Akdis, Karin Hoffmann-Sommergruber, Marek Jutel, Paolo Matricardi, FranÇois Spertin, Nikolai Khaltaev, Jean-Pierre Michel, Laurent Nicod, Peter Schmid-Grendelmeier, Eckard Hamelmann, Thilo Jakob, Thomas Werfel, Martin Wagenmann, Christian Taube, Michael Gerstlauer, Christian Vogelberg, Jean Bousquet, Torsten Zuberbier

**Affiliations:** 1 *Zentrum für Rhinologie und Allergologie, Wiesbaden, *; 2 *Sektion Rhinologie und Allergologie, Klinik für Hals-, Nasen-und Ohrenheilkunde, Universitätsklinikum Gießen und Marburg, Philipps-Universität Marburg, *; 3 *Comprehensive Allergy Centre Charité, Klinik für Dermatologie, Venerologie und Allergologie, Charité – Universitätsmedizin Berlin, *; 4 *Klinik und Poliklinik für Dermatologie und Allergologie, Universitätsklinikum Bonn, *; 5 *III. Medizinische Klinik und Poliklinik Hämatologie, Internistische Onkologie und Pneumologie, Universitätsmedizin Mainz, *; 6 *Zentrum Allergie und Umwelt (ZAUM) Technische Universität und Helmholtz Zentrum München, *; 7 *Klinik und Poliklinik für Dermatologie und Allergologie der Technischen Universität München, *; 8 *Institut für klinische Epidemiologie und Biometrie, Julius-Maximilian-Universität, Würzburg, *; 9 *Allergie- und Asthma-Zentrum Westend, Berlin, *; 10 *Klinik für Pädiatrie m.S. Pneumologie, Immunologie und Intensivmedizin, Charité – Universitätsmedizin Berlin, *; 11 *Abteilung Dermatologie & Allergologie, RWTH Aachen Universität, *; 12 *Medizinische Fakultät der Universität zu Köln, *; 13 *CRI – Clinical Research International Ltd., Hamburg, *; 14 *ClinCompetence Cologne GmbH, Köln, *; 15 *Hautklinik, Universitätsmedizin, Johannes Gutenberg-Universität, Mainz, *; 16 *Europäische Vereinigung für Vitalität und Aktives Altern, Leipzig, *; 17 *Abteilung für Pneumologie, LungenClinic Grosshansdorf, *; 18 *Klinik für Allergologie, Johanniter-Krankenhaus im Fläming Treuenbrietzen GmbH, Treuenbrietzen, *; 19 *Klinik für Innere Medizin Schwerpunkt Pneumologie, Philipps-Universität Marburg, *; 20 *Einheit für Klinische Allergologie (EKA), Helmholtz Zentrum München, Deutsches Forschungszentrum für Gesundheit und Umwelt (GmbH), Neuherberg, *; 21 *Praxis für Dermatologie, Immunologie und Allergologie, Erfurt, *; 22 *Ärzteverband Deutscher Allergologen, Dreieich, *; 23 *Haut- und Laserzentrum an der Oper, München, *; 24 *Academia: München, *; 25 *HNO-Klinik des Klinikums rechts der Isar, Technische Universität München, *; 26 *Zentrum Allergie und Umwelt München (ZAUM); Helmholtz Zentrum München, *; 27 *Praxis für Dermatologie und Allergologie, Münster, *; 28 *Klinik für Hals-, Nasen- und Ohrenheilkunde, Universität Tübingen, *; 29 *Asthma und Allergiezentrum Leverkusen, *; 30 *Klinik für Kinder- und Jugendmedizin, Universitätsklinikum Carl Gustav Carus, Dresden, *; 31 *Praxis für Kinderpenumologie/Allergologie am Kinderzentrum Dresden (Kid), Dresden, *; 32 *Klinik für Dermatologie, Universität Freiburg, *; 33 *Hals-, Nasenund Ohrenarzt, Nordrhein-Westfalen, *; 34 *Klinik für Allergologie, Berufsdermatologie und Umweltmedizin, Universitätsklinikum Münster, *; 35 *Klinik für Dermatologie, Venerologie und Allergologie, Universitätsklinikum, Georg-August-Universität, Göttingen, *; 36 *Klinische Abteilung für Allgemeine HNO, Medizinische Universität Graz, Österreich, *; 37 *Universitätsklinik für Dermatologie und Venerologie, Medizinische Universität Graz, Österreich, *; 38 *Global Allergy and Airways Patient Platform GAAPP, Wien, Österreich, *; 39 *Praxis für Hals-, Nasen- und Ohrenkrankheiten, Wien, Österreich, *; 40 *Floridsdorfer Allergiezentrum, Wien, Österreich, *; 41 *Universitätsklinik für Hals-, Nasen- und Ohrenkrankheiten, Medizinische Universität Wien, Österreich, *; 42 *Institut für Komparative Medizin, Interdisziplinäres Messerli Forschungsinstitut, Veterinärmedizinische Universität Wien und Medizinische Universität Wien, Österreich, *; 43 *Institut für Pathophysiologie und Allergieforschung, Medizinische Universität Wien, Österreich, *; 44 *Abteilung für Atmungs- und Lungenkrankheiten, Krankenhaus Hietzing, Wien, Österreich, *; 45 *Universitätsklinik für Innere Medizin, Medizinische Universität Graz, Österreich, *; 46 *Institut für Pathophysiologie, Medizinische Universität Wien, Österreich, *; 47 *Swiss Institute of Allergy and Asthma Research (SIAF), University of Zurich, Davos, Schweiz, *; 48 *Department of Clinical Immunology, Medizinische Universität Breslau, Polen, *; 49 *Charité – Universitätsmedizin Berlin, *; 50 *Division of Allergy and Immunology, Centre Hospitalier Universitaire Vaudois, Lausanne, Schweiz, *; 51 *GARD Chairman, Genf, Schweiz, *; 52 *Department of Rehabilitation and Geriatrics, University of Geneva, Genf, Schweiz, *; 53 *Clinique Cecil, Hirslanden Gruppe, Lausanne, Schweiz, *; 54 *Abteilung Pneumologie, Centre hospitalier universitaire vaudois, Lausanne, Schweiz, *; 55 *Allergiestation, Dermatologische Klinik, Universitätsspital Zürich, Schweiz, *; 56 *Kinderzentrum Bethel, Evangelisches Klinikum Bethel, Universitätsmedizin OWL der Universität Bielefeld, *; 57 *Klinik für Dermatologie, Allergologie, Universitätsklinikum Gießen, UKGM, Justus-Liebig-Universität Gießen, *; 58 *Klinik fürDermatologie, Allergologie und Venerologie Medizinische Hochschule Hannover, 59HNO-Klinik, Universitätsklinikum Düsseldorf, *; 59 *Klinik für Pneumologie, Ruhrlandklinik, Universitätsmedizin Essen, *; 60 *MACVIA-France, Fondation partenariale FMC VIA-LR, Montpellier, Frankreich, *; 61 *INSERM U 1168, VIMA: Ageing and Chronic Diseases Epidemiological and Public Health Approaches, Villejuif, *; 62 *Université Versailles St-Quentin-en-Yvelines, UMR-S 1168, Montigny le Bretonneux, Frankreich, *; 63 *Euforea, Brussels, Belgien, *; 64 *Charité, Universitätsmedizin Berlin, Humboldt-Universität zu Berlin, *; 65 *Berlin Institute of Health, Comprehensive Allergy Center, Department of Dermatology and Allergy, Berlin, *; 66 *Abteilung für Kinderpneumologie und Allergologie, Medizinische Universität Augsburg, *; 67 *Klinik für Kinderpneumologie und Allergologie, Universitätsklinikum Carl Gustav Carus, Technische Universität Dresden, *; 68 *Dermatologische Allergologie, Allergie-Centrum-Charité, Klinik für Dermatologie, Venerologie und Allergologie, Charité – Universitätsmedizin Berlin *; 69 *Dermatologische Allergologie, Allergie-Centrum-Charité, Klinik für Dermatologie, Venerologie und Allergologie, Charité – Universitätsmedizin Berlin*; A *German ARIA Group*; B *Austrian ARIA Group*; C *Swiss ARIA Group*; D *German Society for Applied Allergology (AEDA)*; E *German Society for Allergology and Clinical Immunology (DGAKI)*; F *Society for Pediatric Allergology (GPA)*; G *AG Clinical Immunology, Allergology and Environmental Medicine of the DGHNO-KHC*; H *European Academy of Allergy and Clinical Immunology (EAACI)*

**Keywords:** Allergen immunotherapy, COVID-19, SCIT, SLIT, antiviral immunity

## Abstract

No abstract available.

Recommendations for Germany, Austria, and Switzerland in adaptation of the ARIA/EAACI position paper Ludger Klimek et al. Handling of allergen immunotherapy in the COVID-19 pandemic: An ARIA-EAACI statement. Allergy. 2020, doi: https://doi.org/10.1111/all.14336

* The first three authors contributed equally.

**German version published in Allergologie, Vol. 43, No. 5/2020, pp. 165-175** [Table Abbreviation]

## Allergen-specific immunotherapy (AIT) 

Allergen-specific immunotherapy (AIT) is the only causal therapy with a proven long-term benefit in allergic airway diseases, such as allergic bronchial asthma or allergic rhinoconjunctivitis, and other allergic diseases [1]. Since its first description more than a hundred years ago (1911 [2]), AIT has been an established and internationally recognized method for the treatment of allergic immediate-type reactions (type I allergy) and associated diseases. 

AIT induces immune tolerance against a specific, individually relevant allergen [3]. Systematic meta-analyses have confirmed that AIT significantly reduces allergic symptoms and the amount of rescue medication used by patients with allergic asthma [4] and allergic rhinoconjunctivitis [5]. 

This applies to both subcutaneous immunotherapy (SCIT) [6, 7] and sublingual immunotherapy (SLIT) [8]. 

AIT reduces the risk of patients with allergic rhinitis to develop asthma [9, 10]. It is also effective in patients with IgE-mediated food allergy [11, 12] and insect venom allergy [13]. Furthermore, this disease-modifying therapeutic option has been shown to be cost saving [14, 15, 16]. 

Scientific studies on the association between allergic airway diseases and viral airway infections show inconsistent results, and little is known about the effects of AIT on viral airway infections and vice versa [17]. 

In their prospective and comparative clinical study, Ahmetaji et al. [18] found no significant difference in effectiveness or symptom improvement in patients with allergic asthma using SCIT with or without influenza-like viral infections. The laboratory chemical and hematological standard parameters and various cytokines during treatment and 1 year follow-up were also not different [18]. These preliminary data suggest that SCIT is safe and well-tolerated in patients with influenza virus infection. 

Iemoli et al. [19] investigated the safety and clinical efficacy of sublingual grass allergy tablet immunotherapy in a group of HIV-positive patients with allergic rhinitis receiving antiretroviral therapy. HIV infection was considered a relative contraindication for AIT; however, highly active antiretroviral treatment has meanwhile improved immune function and life expectancy of HIV-infected patients so much that an attempt at therapy seems reasonable, especially since the incidence of allergic airway diseases in HIV-infected patients is comparable to that of the general population [19]. Data on clinical efficacy showed a significant improvement of patients treated with SLIT compared to controls, but no significant change in the number of CD4-positive T cells and HIV viral load in both groups was observed. These data show that SLIT can be effective, safe, and well-tolerated in viro-immunologically controlled HIV-positive patients. 

Furthermore, it could be shown that cytomegaloviruses (CMV) increased the allergenic potential of otherwise weak environmental allergens in airway epithelium in a murine model when exposed to CMV and ovalbumin (OVA) [20]. 

In contrast, virus-like particles (VLPs) as modern vaccine components can even be used in the AIT of airborne and food allergens (peanut) in the near future [21, 22]. 

## Coronavirus disease 2019 (COVID-19) 

On March 11, 2020, the World Health Organisation (WHO) declared the pandemic outbreak of an infectious disease defined as “Coronavirus disease 2019 (COVID-19)”. The COVID-19 pandemic is currently spreading around the globe. COVID-19 is caused by a novel strain of human coronaviruses, which the International Committee on Taxonomy of Viruses (ICTV) has named SARS-CoV-2 (Severe acute respiratory syndrome-Coronavirus 2). SARS-CoV-2 war first discovered and identified in a group of pneumonia patients in Wuhan, China, in December 2019 [23, 24]. SARS-CoV-2 is a Betacoronavirus of the subgenus Sarbecovirus and the subfamily Orthocoronavirinae. It can be isolated from human samples obtained from respiratory secretions, nasal and pharyngeal smears and can be isolated on cell cultures [23, 24]. SARS-CoV-2 is the seventh member of the Coronaviridae family that can infect humans. It is covered by a lipid membrane that can be disrupted by detergents and is different from the Middle East respiratory syndrome-related coronavirus (MERS-CoV), severe acute respiratory syndrome coronavirus (SARS-CoV), and the coronaviruses responsible for common cold (229E, OC43, NL63, and HKU1) [25]. Coronaviruses are zoonotic, i.e., they are transmitted between animals and humans. 

COVID-19 infections can be clinically asymptomatic, cause mild illness with or without lung involvement, or lead to the most severe forms of pneumonia and multi-organ failure [26]. 

Frequent symptoms of COVID-19 infections are fever, cough, shortness of breath, and breathing difficulties as well as taste and smell dysfunction. Further signs of viral airway infection can be nasal symptoms and a sore throat. In more severe cases, infection with COVID-19 can lead to pneumonia, severe acute respiratory syndrome, renal failure, and death [26, 27, 28, 29, 30]. In the scientific literature, higher age and comorbidities, such as chronic airway diseases, diabetes mellitus, coronary heart disease, and immunodeficiency of various origin, have been listed as risk factors for severe course, hospitalization, intubation, and death [26, 27, 28, 30]. 

In general, each viral airway disease can exacerbate asthma. Children and adults with bronchial asthma are currently recommended to consistently continue their prescribed long-term therapy with inhaled corticosteroids in order to maintain or achieve the best possible asthma control. Ongoing therapies should not be reduced or discontinued at the moment. 

Since COVID-19 is caused by a newly identified virus strain, no specific antiviral substances or vaccines tested in controlled studies are currently available, and it is believed that there is no pre-existing immunity in the population [31]. In most cases, coronaviruses are transmitted from person to person through large respiratory droplets, by inhalation or deposition on the mucosal surface. Other routes of infection with coronaviruses are: contact with contaminated surfaces, direct person-to-person contact, and inhalation of aerosols originating from sneezing or coughing or dental treatment [32]. SARS-CoV-2 virus has been detected in airway, stool, and blood samples [32]. 

The highest risk of transmission for medical staff is present when standard precautions are missing, when primary infection prevention and control measures for respiratory infections are not undertaken, and when infected, potentially asymptomatic patients who are not yet tested positive for COVID-19 are treated without protective measures. Because of the possible transmission by aerosols [33, 34] in a large number of patients with airway disease, a prudent approach is also recommended in allergy practice [35], where, e.g., lung function measurement, breath tests, and nasal provocation are standard procedures. 

Additional background information on COVID-19 is available online from the European Centre for Disease Prevention and Control (ECDC) [36], the WHO [37], and Rapid Risk Assessment of the ECDC [31]. 

In view of the limited clinical data available, there is no evidence that patients with allergic rhinitis develop additional symptoms or more severe courses as compared to other patients [26, 35]. Allergic children had a mild course, similar to non-allergic children [26]. 

## Immune mechanisms in AIT and COVID-19 – Differences and similarities 

AIT aims at inducing allergen-specific immune tolerance in allergic patients by utilizing effects on several immune mechanisms (Table 1), including T cells, B cells, innate lymphoid cells (ILCs), and effector cells, such as eosinophils, mast cells, and basophils [15, 38]. One of the most important changes evoked by AIT is the development of a regulatory T and B cell response and its suppressive cytokines, like IL-10 or TGF, and surface molecules, such as CTLA-4 and PD1, all of which form an immune-suppressive environment [15, 38]. This immune-regulatory response takes place in targeted antigen/allergen-specific T and B cells, but does not affect the entire immune system and, above all, does not cause any systemic immunodeficiency. T-cell responses in COVID-19 are associated with lymphopenia, which mainly affect memory T lymphocytes. Both CD4 and CD8+ T cells decrease, with this change being more pronounced in CD8+ T cells [15, 38]. In patients with SARS-CoV-2 infection, cytotoxic CD8+ T lymphocytes and NK cells are essential for an adequate antiviral response [39]. A recent study suggests that patients show a functional exhaustion of cytotoxic CD8+ T lymphocytes when SARS-CoV-2 infection is present [39]. The total number of NK and CD8+ cells was markedly reduced in patients with SARS-CoV-2 infection [39]. This can lead to dysfunction of antiviral immunity and play a role in the pathogenesis and severity of COVID-19. 

AIT significantly and highly selectively decreases allergen-specific Th2 cells in the blood and reduces the general type 2 response. 

## Preventive measures in allergy centers/practices and control measures in AIT 

We recommend the use of measures for infection prevention and control in all patients undergoing AIT, in accordance with ECDC and WHO. These measures can be interpreted and applied differently in individual regions or countries, which is why all regional and national guidelines should be followed for infection prevention, including the measures contained in this document and the procedures for reporting and transferring examined patients and probable/confirmed COVID-19 cases. 

Patients with typical airway symptoms should be advised to resort to telephone/e-health/telemedicine/online consultation instead of coming to the practice or allergy center [36, 40] (triage). This will reduce the number of people with COVID-19 symptoms that have contact with the professional staff. 

The staff, including doctors, medical assistants, nutritional scientists, nursing and administrative staff, and all other staff at the facility with patient contact, should be made aware of: a) the current epidemiological situation of COVID-19 in Germany/Austria/Switzerland and worldwide; b) known risk factors for infection; c) clinical signs and symptoms of COVID-19; d) recommended measures to prevent and contain infections in their region or country, including those mentioned in this document; e) procedures for reporting and transferring examined patients and probable/confirmed cases taking into account the appropriate regional regulations and specifications [36, 40]. 

They should be continuously and regularly instructed regarding the current measures that are recommended especially for medical staff and that need to be taken when they develop symptoms themselves or have contact with SARS-CoV-2-positive patients. Suitable personal protective equipment (PPE) should be available for all staff at the treating facility to ensure standard, contact, and droplet protection. 

In each allergy practice/center, one person (e.g., senior consultant, nurse, medical assistant) should lead the COVID-19 preventive measures and implement the corresponding guidelines for infrastructure and control measures. 

Signs indicating the main symptoms suggestive of a COVID-19 infection (fever, cough, dyspnea, etc.) should be posted at all entrance doors, and visitors showing one of these symptoms should be advised not to enter the practice/allergy department. 

The staff and all persons entering a practice/allergy department should take suitable measures for hand hygiene using soap and water or an alcohol-based hand disinfectant with an antiviral effect. 

Based on a case-by-case risk assessment, the use of PPE should be considered in the AIT setting. With the current knowledge of COVID-19 transmission, in which larger liquid droplets appear to play a significant role (although airborne transmission cannot be ruled out at the present time), and taking into account the possible lack of PPE in healthcare facilities as the number of COVID-19 patients increases, the proposed PPE set for droplet, contact, and airborne transmission (gloves, eye protection, gown, and FFP2/FFP3 respiratory protection mask) can be adapted for the clinical assessment of suspected COVID-19 cases. 

If available, a nose and mouth mask (surgical mask or surgical mouth and nose protection) should be provided for patients with respiratory symptoms (e.g., cough) [41]. 

Only one person should accompany children and adolescents who must also apply all hygienic measures. 

Staff carrying out aerosol-generating examinations, e.g., provocation tests [41], should wear the proposed PPE set to prevent droplet, contact, and airborne transmission (gloves, eye protection, gown, and FFP2/FFP3 respiratory protection) [42]. If this is not possible, these tests should currently not be carried out. 

To maximize PPE use staff should be assigned to perform procedures in dedicated areas if supply is insufficient [43]. 

## Managing AIT during the COVID-19 pandemic 

AIT requires repeated contact between patient and physician/medical assistant/nurse over a longer period of time, e.g., 3 years. 

In SCIT, injections are given daily, weekly (updosing phase), or every 4 – 8 weeks (maintenance phase). 

SLIT is initiated in allergy practices or centers, then the therapy is continued by the patient with regular follow-ups by the treating physician. 

Each SCIT or SLIT product must be approved by the responsible authorities and must contain instructions for use for patients, physicians/allergists, and nurses. For most products authorized in Europe, the instructions for use recommend that patients with acute respiratory infection should temporarily discontinue AIT until the infection has completely resolved. We recommend to take similar measures during the current COVID-19 pandemic. In confirmed cases, AIT (both SCIT and SLIT) should be discontinued, independently of the severity of the disease, until the symptoms have completely resolved. 

The start of AIT in allergic patients without known SARS-CoV-2 exposure who do not show COVID-19 symptoms, but whose current SARS-CoV-2 infection and immune status are unknown, requires a thorough examination for signs of SARS-CoV-2 infection during the indication for AIT, and the same examination should be repeated at the start of AIT. The delivery times of the AIT preparations and the risk of infection due to the patient’s current personal and professional environment must be taken into account. 

On the one hand, SLIT offers the possibility of taking the allergen preparation at home without further medical supervision after the first dose had been given in the practice/allergy department. This avoids repeated visits to the practice/allergy department, which is associated with an infection risk. On the other hand, the need to discontinue AIT can better be assessed by a physician, as patients may not be able to correctly correlate their symptoms to a potential COVID-19 infection. Subclinical/oligosymptomatic courses represent a particular challenge for patients on self-medication. Thus, patients taking SLIT at home should be advised to contact the treating physician before applying the next dose if signs of infection occur. 

When using SCIT preparations, the injection intervals can be prolonged. Patients who have recovered from COVID-19 or in whom an adequate SARS-CoV-2 antibody reaction is detected after (possibly asymptomatic) disease [37] can start or continue AIT as planned [35]. AIT can also be continued as usual in patients without clinical signs and symptoms of COVID-19 or other infections who did not travel to regions with COVID-19 cases (high-risk regions) within the previous 14 days. [Table Box1][Table Box2]

## Conflict of interest 

R. Buhl: Lectures for and/or consultancy of AstraZeneca, Boehringer Ingelheim, Chiesi, Cipla, Novartis, Roche, Sanofi, and Teva; Research support for Universitätsmedizin Mainz: Boehringer Ingelheim, GlaxoSmithKline, Novartis und Roche – unrelated to this paper. 

R. Brehler: Lectures for ALK, Allergopharma, Almirall, AstraZeneca, Bencard, Gesellschaft zur Förderung der Dermatologischen Forschung und Fortbildung, Gesellschaft für Information und Organisation, GSK, Dr. Pfleger, HAL, Leti, Merck, Novartis, Oto-Rhino-Laryngologischer Verein, Pierre Fabre, Pohl Boskamp, Stallergenes, Thermo-Fischer; Consultancy for Allergopharma, Bencard, HAL, Leti, Novartis; Clinical studies for Allergopharma, Bencard, Biotech Tools, Genentech, Leti, Novartis, Circassia – unrelated to this paper. 

U. Darsow was lecturer, principal investigator, and consultant for ALK Abello, Bencard, and Novartis Pharma – unrelated to this paper. 

T. Jakob received grants, personal fees, or non-financial support from Novartis, ALK-Abelló, Bencard/Allergy Therapeutics, Allergopharma, Thermo Fisher Scientific, and Celgene – unrelated to this paper. 

M. Jutel: Personal fees from ALKAbello, Allergopharma, Stallergenes, Anergis, Allergy Therapeutics, Circassia, Leti, Biomay, and HAL – during the conduct of this study; Personal fees from AstraZeneka, GSK, Novartis, Teva, Vectura, UCB, Takeda, Roche, Janssen, Medimmune, and Chiesi – unrelated to this paper. 

L. Klimek: Grants and/or personal fees from Allergopharma, MEDA/ Mylan, HAL Allergie, ALK Abelló, Leti, Stallergenes, Quintiles, Sanofi, ASIT biotech, Lofarma, Allergy Therapeut., Astra- Zeneca, GSK, Inmunotk – unrelated to this paper; Member of the following organizations: AeDA, DGHNO, Deutsche Akademie für Allergologie und klinische Immunologie, HNO-BV GPA, EAACI. 

S. Lau: Consultant for Allergopharma. 

P. Matricardi: Grants and/or personal fees from DFG, Hycor, Omron, Stallergens, Euroimmun, Novartis, TPS, Stallergenes- Greer – unrelated to this paper; Non-financial support from Thermo Fisher Scientific – unrelated to this paper. 

O. Pfaar reports to have received research grands and/or personal fees from ALK-Abelló, Allergopharma, Stallergenes Greer, HAL Allergy Holding B.V./HAL Allergie GmbH, Bencard Allergie GmbH/ Allergy Therapeutics, Lofarma, Biomay, Circassia, ASIT Biotech Tools S.A., Laboratorios LETI/LETI Pharma, MEDA Pharma/ MYLAN, Anergis S.A., Mobile Chamber Experts (a GA2LEN Partner), Indoor Biotechnologies, Glaxo Smith Kline, Astellas Pharma Global, EUFOREA, Roxall, Novartis, Sanofi Aventis, Med Update Europe GmbH und streamedup! GmbH for the past 36 months – unrelated to this paper. 

R. Valenta: Research grants from Viravaxx, Vienna, Austria, and HVD Life Sciences, Vienna, Austria; Consultant for Viravaxx. 

M. Worm: Personal and/or consultancy fees from ALK-Abelló Arzneimittel GmbH, Mylan Deutschland GmbH, Leo Pharma GmbH, Sanofi-Aventis Deutschland GmbH, Regeneron Pharmaceuticals, DBV Technologies SA, Stallergenes GmbH, HAL Allergie GmbH, Allergopharma GmbH & Co.KG, Bencard Allergie GmbH, Aimmune Therapeutics UK Limited, Actelion Pharmaceuticals Deutschland GmbH, Novarits AG, and Biotest AG. 

M. Wagenmann: Research grants and/or personal fees from ALKAbelló, Allergopharma, AstraZeneca, Bencard, Genzyme, GlaxoSmithKline, HAL Allergie, LETI Pharma, MEDA Pharma, Novartis, Sanofi Aventis, Stallergenes, and Teva. 

T. Werfel: Adboards, payed lectures for für ALK Scherax, Bencard, Leti, and Stallergens. 

T. Zuberbier: Consultant for Bayer Health Care, FAES, Novartis, and Henkel; Research grants from Novartis and Henkel; Lecture fees from AstraZeneca, AbbVie, ALK, Almirall, Astellas, Bayer Health Care, Bencard, Berlin Chemie, FAES, HAL, Leti, Meda, Menarini, Merck, MSD, Novartis, Pfizer, Sanofi, Stallergenes, Takeda, Teva, UCB, Henkel, Kryolan, and L’Oréal – unrelated to this paper. 

W. Aberer, C. Akdis, M. Akdis, S. Becker, K.-C. Bergmann, T. Bieber, T. Biedermann, J. Bousquet, J. Buters, A. Chaker, W. Czech, A. Fink-Wagner, T. Fuchs, M. Gerstlauer, E. Hamelmann, K. Hoffmann-Sommergruber, F. Horak, K. Jung, T. Keil, N. Khaltaev, J. Kleine-Tebbe, M. Maurer, H. Merk, J.-P. Michel, R. Mösges, N. Mülleneisen, K. Nemat, L. Nicod, V. Niederberger- Leppin, I. Pali- Schöll, W. Pohl, K. Rabe, U. Rabe, J. Ring, R. Roller-Wirnsberger, J. Saloga, W. Schlenter, P. Schmid-Grendelmeier, F. Spertini, O. Spranger, P. Staubach, P. Stute, C. Taube, P.- V. Tomazic, C. Vogelberg, C. Vogelmeier, W. Wehrmann, S. Wöhrl, and H. Wrede declare that they have no conflicts of interest.****

**Abbreviations Abbreviation:** Abbreviations

AIT	Allergen-specific immunotherapy
ARIA	Allergic rhinitis and its impact on asthma
CD	Cluster of differentiation
CMV	Cytomegalovirus
CTL	Cytotoxic T lymphocytes
CTLA-4	Cytotoxic T lymphocyte antigen-4
EAACI	European Academy of Allergy and Clinical Immunology
FFP2/FFP3	Filtering facepiece (surgical mask, protection class 2/protection class 3)
ICTV	International Committee on Taxonomy of Viruses
IgE	Immunoglobulin E
IgG	Immunoglobulin G
IgM	Immunoglobulin M
IL	Interleukin
ILC	Innate lymphoid cell
MERS-CoV	Middle east respiratory syndrome coronavirus
NK	Natural killer cells
OVA	Ovalbumin
PD-1	Programmed cell death protein
PCR	Polymerase chain reaction
SARS-CoV-2	Severe acute respiratory syndrome coronavirus 2
SCIT	Subcutaneous immunotherapy
SLIT	Sublingual immunotherapy
TGF	Transformig growth factor
Th	T helper cell
Treg	Regulatory T cells
VLP	Virus-like particles
WHO	World Health Organization

**Table 1.** Immunologic phenomena in AIT and COVID-19 (from [35]).

Immunologic changes	AIT	COVID-19
T cell responses	Suppression of Th2 cells, induction of Treg and Th1 cells. No reduction of total number of lymphocytes in peripheral blood	Lymphopenia in severe cases
CD8+ T cells	No major change	Severe lymphopenia has been observed in CD8+ T cells
Th1-Th2 responses	AIT decreases allergen-specific Th2 responses in circulation and in affected organs (e.g., nose)	Severe disease shows systemic severe inflammatory response with cytokine storm
Eosinophils	Decrease in their numbers and eos-specific mediators in the nose	Systemic decrease in their numbers in more than half of the patients
Specific antibody levels	Allergen-specific IgE decreases later in the course, with an early increase in specific IgG4	In the acute phase, virus-specific IgM increases followed by virus-specific IgG during and after convalescence (seroconversion)

AIT = Allergen-specific immunotherapy; eos = eosinophilic granulocyte; Ig = immunoglobulin; Th = T helper cell; Treg = regulatory T cells.

**Box 1 Box1:** Recommendations for AIT in non-infected individuals during the COVID-19 pandemic or in recovered patients after COVID-19 infection (modified from [35]).

Termination of subcutaneous immunotherapy is not generally necessary. Particularly in potentially life-threatening allergies, such as insect venom allergy, SCIT should be continued taking into account the risk-benefit analysis. It can be considered to extend the injection intervals according to the summary of product characteristics of the respective therapeutic allergen.
Termination of sublingual immunotherapy is not generally necessary. Patients should be supplied with SLIT preparations sufficient to last for a minimum of 14 days in a quarantine situation.
In the current COVID-19 pandemic, both SCIT and SLIT can be continued in asymptomatic patients with negative PCR tests, in patients without know exposure or contact with SARS-CoV-2-positive people, and in patients who did not travel to high-risk regions or were adequately quarantined after traveling.
To start SCIT or SLIT in allergic patients without known SARS-CoV-2 exposure and without COVID-19 symptoms, a thorough medical history and examination for signs of infection are required at the start of the treatment and with every further SCIT injection or SLIT administration. The risk of infection from the patient’s personal and professional environment must be taken into account.
Practices and allergy centers must be prepared for the current COVID-19 pandemic. Recommendations of the WHO and national and regional authorities should be followed.
These recommendations should be continuously updated and adapted to new scientific findings and recommendations made by authorities.

**Box 2 Box2:** Recommendations for AIT in patients with diagnosed or suspected SARS-CoV-2 infection (modified from [35]).

(Temporary) discontinuation of SCIT is recommended for patients with positive SARS-CoV-2 lab test (SARS-CoV-2 detection using PCR or IgM test).
(Temporary) discontinuation of SLIT is recommended for patients with positive SARS-CoV-2 lab test (SARS-CoV-2 detection using PCR or IgM test).
In symptomatic patients with suspected SARS-CoV-2 infection and sufficient contact with positive patients and/or history of traveling to high-risk areas, both SCIT and SLIT should be discontinued until an adequate quarantine period has been completed.
